# Burden of Type 2 Diabetes and Associated Cardiometabolic Traits and Their Heritability Estimates in Endogamous Ethnic Groups of India: Findings From the INDIGENIUS Consortium

**DOI:** 10.3389/fendo.2022.847692

**Published:** 2022-04-14

**Authors:** Vettriselvi Venkatesan, Juan Carlos Lopez-Alvarenga, Rector Arya, Deepika Ramu, Teena Koshy, Umarani Ravichandran, Amaresh Reddy Ponnala, Surendra K. Sharma, Sailesh Lodha, Krishna K. Sharma, Mahaboob Vali Shaik, Roy G. Resendez, Priyanka Venugopal, Parthasarathy R, Noelta Saju, Juliet A. Ezeilo, Cynthia Bejar, Gurpreet S. Wander, Sarju Ralhan, Jai Rup Singh, Narinder K. Mehra, Raghavendra Rao Vadlamudi, Marcio Almeida, Srinivas Mummidi, Chidambaram Natesan, John Blangero, Krishna M. Medicherla, Sadagopan Thanikachalam, Thyagarajan Sadras Panchatcharam, Dileep Kumar Kandregula, Rajeev Gupta, Dharambir K. Sanghera, Ravindranath Duggirala, Solomon F. D. Paul

**Affiliations:** ^1^ Department of Human Genetics, Faculty of Biomedical Sciences and Technology, Sri Ramachandra Institute of Higher Education and Research (Deemed to be University), Chennai, India; ^2^ Department of Human Genetics and South Texas Diabetes and Obesity Institute, University of Texas Rio Grande Valley, Brownsville, TX, United States; ^3^ Department of Medicine, Rajah Muthiah Medical College Hospital, Annamalai University, Chidambaram, India; ^4^ Department of Endocrinology, Krishna Institute of Medical Sciences (KIMS) Hospital, Nellore, India; ^5^ Department of Endocrinology, Galaxy Specialty Centre, Jaipur, India; ^6^ Departments of Preventive Cardiology, Internal Medicine and Endocrinology, Eternal Heart Care Centre and Research Institute, Mount Sinai New York Affiliate, Jaipur, India; ^7^ Department of Pharmacology, Lal Bahadur Shastri College of Pharmacy, Rajasthan University of Health Sciences, Jaipur, India; ^8^ Department of Endocrinology, Narayana Medical College and Hospital, Nellore, India; ^9^ Department of Pediatrics, College of Medicine, University of Oklahoma Health Sciences Center, Oklahoma City, OK, United States; ^10^ Hero Dayanand Medical College (DMC) Heart Institute, Dayanand Medical College and Hospital, Ludhaina, India; ^11^ Honorary or Emeritus Faculty, Central University of Punjab, Bathinda, India; ^12^ Honorary or Emeritus Faculty, All India Institute of Medical Sciences and Research, New Delhi, India; ^13^ Honorary or Emeritus Faculty, Genome Foundation, Hyderabad, India; ^14^ Department of Biotechnology, Birla Institute of Scientific Research, Jaipur, India; ^15^ Department of Cardiology, Sri Ramachandra Medical College and Research Institute, Sri Ramachandra Institute of Higher Education and Research (Deemed to be University), Chennai, India; ^16^ Chancellor, Avinashilingam University, Coimbatore, India; ^17^ Department of Physiology, College of Medicine, University of Oklahoma Health Sciences Center, Oklahoma City, OK, United States; ^18^ Department of Pharmaceutical Sciences, College of Pharmacy, University of Oklahoma Health Sciences Center, Oklahoma City, OK, United States

**Keywords:** type 2 diabetes, cardiometabolic traits, Indian population, epidemiology, genetic epidemiology, family study, heritability

## Abstract

To assess the burden of type 2 diabetes (T2D) and its genetic profile in endogamous populations of India given the paucity of data, we aimed to determine the prevalence of T2D and estimate its heritability using family-based cohorts from three distinct Endogamous Ethnic Groups (EEGs) representing Northern (Rajasthan [Agarwals: AG]) and Southern (Tamil Nadu [Chettiars: CH] and Andhra Pradesh [Reddys: RE]) states of India. For comparison, family-based data collected previously from another North Indian Punjabi Sikh (SI) EEG was used. In addition, we examined various T2D-related cardiometabolic traits and determined their heritabilities. These studies were conducted as part of the Indian Diabetes Genetic Studies in collaboration with US (INDIGENIUS) Consortium. The pedigree, demographic, phenotypic, covariate data and samples were collected from the CH, AG, and RE EEGs. The status of T2D was defined by ADA guidelines (fasting glucose ≥ 126 mg/dl or HbA1c ≥ 6.5% and/or use of diabetes medication/history). The prevalence of T2D in CH (N = 517, families = 21, mean age = 47y, mean BMI = 27), AG (N = 530, Families = 25, mean age = 43y, mean BMI = 27), and RE (N = 500, Families = 22, mean age = 46y, mean BMI = 27) was found to be 33%, 37%, and 36%, respectively, Also, the study participants from these EEGs were found to be at increased cardiometabolic risk (e.g., obesity and prediabetes). Similar characteristics for the SI EEG (N = 1,260, Families = 324, Age = 51y, BMI = 27, T2D = 75%) were obtained previously. We used the variance components approach to carry out genetic analyses after adjusting for covariate effects. The heritability (h^2^) estimates of T2D in the CH, RE, SI, and AG were found to be 30%, 46%, 54%, and 82% respectively, and statistically significant (P ≤ 0.05). Other T2D related traits (e.g., BMI, lipids, blood pressure) in AG, CH, and RE EEGs exhibited strong additive genetic influences (h^2^ range: 17% [triglycerides/AG and hs-CRP/RE] - 86% [glucose/non-T2D/AG]). Our findings highlight the high burden of T2D in Indian EEGs with significant and differential additive genetic influences on T2D and related traits.

## Introduction

Type 2 diabetes (T2D) is a complex blood glucose-homeostasis disorder characterized by both insulin resistance and pancreatic β-cell dysfunction (1). The compound burden of an increasing global T2D epidemic together with its comorbid conditions such as obesity, hypertension, and cardiovascular disease (CVD) has become a major global public health problem, particularly in countries such as China, India, and the United States (US) (2–5). Indeed, Asia including the Indian subcontinent has become the epicenter of the escalating diabetes epidemic; currently, India has the second highest number of people affected with diabetes worldwide next to China (4–6). According to the International Diabetes Federation (IDF), an estimated 77 million people (20-79 years) have diabetes in India in 2019, which is projected to be 101 million people in 2030 and ~134 million people in 2045, respectively (4). Given its unique population genetic background and cultural history, the contemporary Indian population is composed of numerous sub-populations (e.g., tribal vs. caste [from now onwards referred at as Endogamous Ethnic Groups/EEGs] groups) with remarkable cultural, linguistic, regional, and genetic diversity (7–9).

Numerous epidemiological studies, local and national, have shown that the occurrence of T2D exhibits remarkable variation by geography (rural vs. urban and Northern vs. Southern regions of India) and socio-economic status (3, 5, 10–18). For example, the national Indian Council of Medical Research (ICMR)-INdia DIABetes (INDIAB) population-based study involving 15 states of India estimated the prevalence of T2D to be 7.3%; and, it varied by state/region, ranging from 4.3% in Bihar to 10.0% in Punjab and was higher in urban areas than in rural areas (3). Uniquely, Indian populations (and other South Asian [SA] populations), compared with other populations, are at increased risk for the development of T2D at younger ages and at lower body mass index (BMI) levels (18–22). It is shown that Indians have increased levels of insulin resistance and a stronger genetic predisposition to T2D (23–27).

Although India represents nearly one fifth of the global population, there have only been a few genome-wide association studies (GWASs) of T2D involving populations in India including our own study of the Punjabi Sikh population or immigrant populations of Indian ancestry, which localized a few T2D susceptibility loci (28–32). In addition, there is a paucity of data on EEG specific family-based genetic epidemiological studies (26, 33–37). In general, Indian populations are ideal to conduct genetic epidemiologic investigations of complex diseases such as T2D and obesity, given their high levels of endogamy, large family structures, historic admixture patterns mirroring a North-South gradient (Ancestral North Indians vs. Ancestral South Indians), and a high degree of genetic differentiation among them reflecting the importance of local biocultural backgrounds (38–42).

Therefore, the purpose of the current study is to compare the epidemiological (e.g., phenotype differences) and genetic epidemiological (e.g., genetic and environmental influences) profiles among four EEGs based on pedigree-based data sets, two representing north Indian states of Punjab (data already available) and Rajasthan and other two representing the south Indian states of Tamil Nadu and Andhra Pradesh. It should be noted that, in addition to the determination of overall genetic and environmental influences on a given phenotype based on pedigree information, compared to population-based studies, pedigree-based studies provide several advantages to the identification of rare variation, the main advantage being that rarer variants (with larger effect sizes) will be present at a much higher frequency than in the general population (43, 44). Our follow-up studies will assess the extent to which the common and rare variants to be found through targeted sequencing of selected SA-specific T2D risk loci including our own GWAS T2D signal found in the Sikh population are transportable to other three EEGs to be examined in this study. Thus, here we report the findings of T2D burden in the families of the selected EEGs and genetic and environmental influences on T2D and its related cardiometabolic phenotypes.

## Materials and Methods

### Indian Diabetes Genetic Studies in Collaboration With US (INDIGENIUS) Consortium

As part of the joint Indian Council of Medical Research (ICMR) and National Institute of Diabetes and Digestive and Kidney Diseases/National Institutes of Health (NIDDK/NIH), US, Collaborative Research Partnership (CRP), we developed a new Indo-US bilateral CRP on genetics of diabetes research. The research activities of this study were initiated after obtaining the project-specific (i.e., Indian and US Institutions) Institutional Review Board (IRB) approvals in accordance with both the Government of India and the US regulations for the protection of human subjects as well as institution-specific collaborative research policies. Prior to the conduct of the study, a workshop/symposium involving the Indian and US investigators was conducted at Sri Ramachandra Institute for Higher Education and Research (SRIHER), Chennai to present and discuss the country-specific protocols, standardization of research procedures and conduct, and the overall collaborative structure to establish the INDIGENIUS Consortium.

### Study Design, Populations (i.e., EEGs), and Recruitment

**Figure 1:**
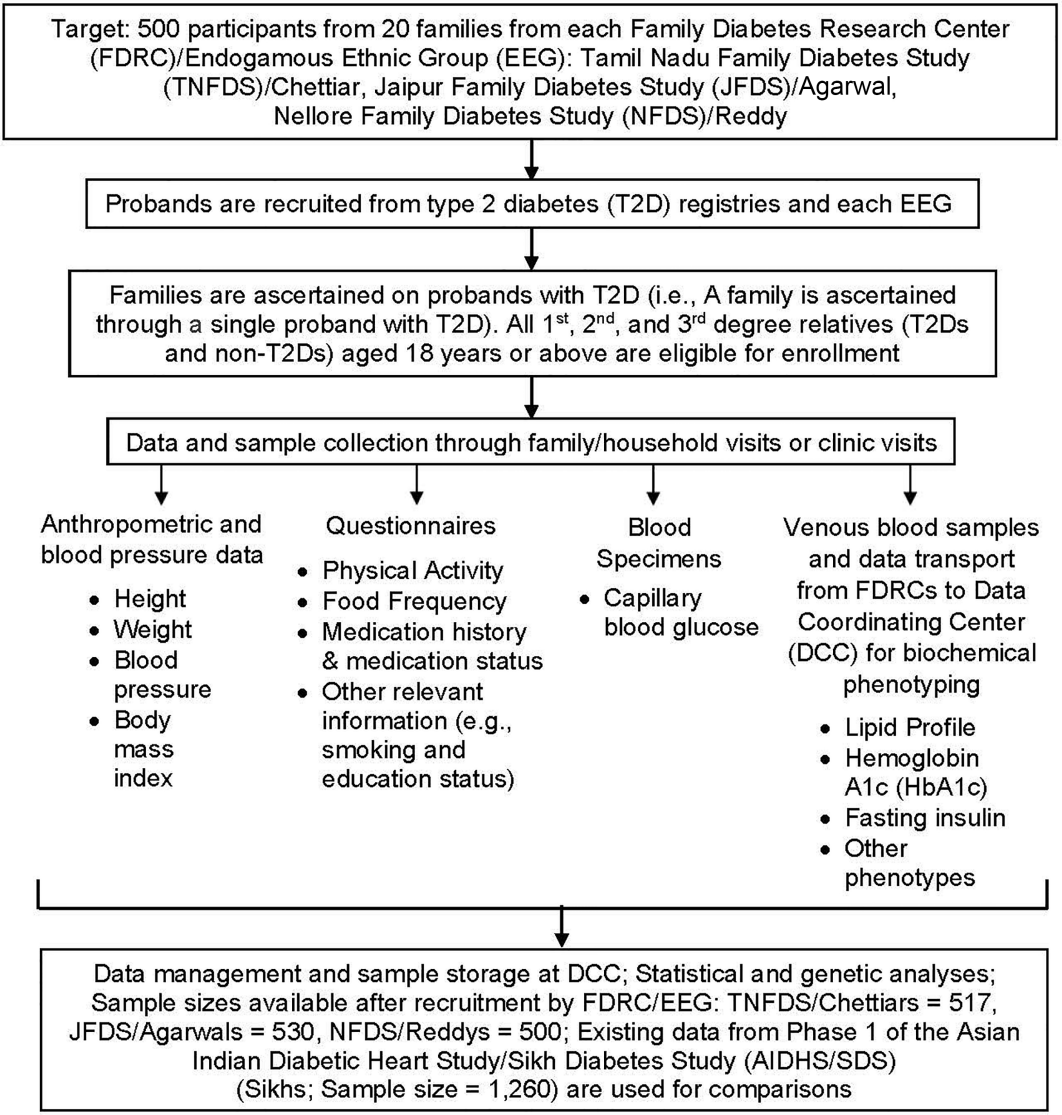
Flow chart depicting study design and methodological tools including recruitment, data collection, and analyses.

The study design was developed jointly by the Indian and US investigators. As part of the ICMR project, three independent Family Diabetes Research Centers (FDRCs), the Tamil Nadu Family Diabetes Study (TNFDS), Chennai, Tamil Nadu (South India); the Jaipur Family Diabetes Study (JFDS), Jaipur, Rajasthan (Northwest India); and the Nellore Family Diabetes Study (NFDS), Nellore, Andhra Pradesh (South India) were established. The FDRC at SRIHER, Chennai has served as the Data Coordinating Center (DCC). Each of the FDRCs recruitment goals were 500 individuals from ~20 large families; and the families were ascertained on probands that were previously identified as having T2D based on medical records or information from existing case registries available at the FDRCs. The study design and methodological tools used are summarized in **Figure 1**.

The planned sample size of 500 individuals per EGG corresponds to the general target heritability of 0.20. After the actual recruitment, we found that the following heritability estimates per EEG/pedigree structures could be detected at 80% power: TNFDS = 0.210, NFDS = 0.215, and JFDS = 0.163. Our sampling strategy was to recruit study participants in random order without any attempt to recruit multiplex families preferentially. Thus, a family from each EEG was ascertained through a single proband with T2D. The T2D probands in this study constitute a community-based case series of T2D, representing a community-based sample of pedigrees. Once a family was identified for recruitment, all 1^st^, 2^nd^, and 3^rd^ degree relatives (T2Ds and non-T2Ds), aged from 18 years or above (i.e., adults), living in a household and its surroundings were invited to participate in a given FDRC study. In addition, every effort was made to recruit family members away from homes as much as possible. Children aged 17 years and below were not recruited for this study. All of the field activities including family member recruitment; collection of demographic, phenotypic and covariate data; and, collection of blood specimens were performed under the direct supervision of the primary investigators of a given FDRC, who were assisted by the research assistants and/or the clinical staff.

**Figure 2:**
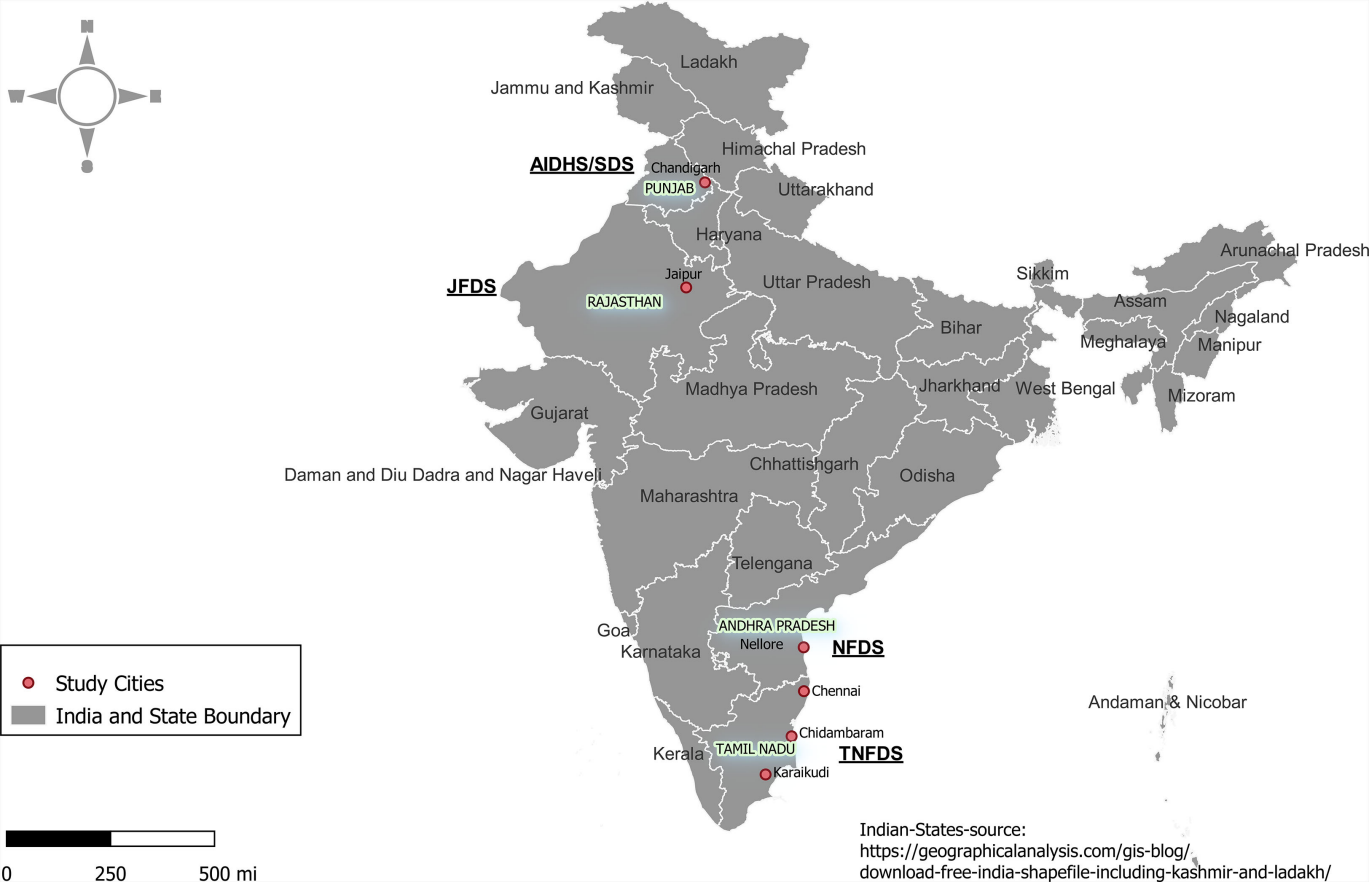
The geographic locations of the study sites in the states of Andhra Pradesh, Punjab, Rajasthan, and Tamil Nadu in India.

The JFDS recruited families from the Agarwal EEG, one of the largest business communities in India found throughout northern India including the state of Rajasthan (45). The NFDS recruited families from the Reddy EEG, one of the dominant farming communities composed of wealthy landowners, businessmen, and people in other professions including government jobs mainly inhabited in the state of Andhra Pradesh (46). The TNFDS recruited families from the Chettiar EEG, a sub-group of the Tamil population originating from Chettinad in Tamil Nadu (47). Chettinadu literally means Chettiars’ state. It is a community of traders and financiers for many centuries. The EEG identity was self-declared by study participants with information supported by parental and grandparental native backgrounds. The data from a Sikh Khatri EEG from the state of Punjab were already collected and used in this study for the purpose of comparisons. Through NIH support, over 4,700 individuals were recruited as part of the Asian Indian Diabetic Heart Study/Sikh Diabetes Study [AIDHS/SDS], and the details of these studies were reported previously (34, 36, 48). Briefly, the Sikh Khatri EEG family study (Phase I of the AIDHS/SDS) was comprised of 1,260 individuals distributed across 340 families (mostly nuclear in nature) for whom demographic, phenotypic/covariate data, and blood samples were already available (30, 34, 36, 49, 50). The Agarwal and Sikh EEGs are speakers of Indo-Europeans languages, and the Reddy and Chettiar EEGs are speakers of Dravidian languages. The geographic locations of the study sites are depicted in **Figure 2**.

### Phenotypic and Covariate Data Collection

The pedigree, demographic, phenotypic, environmental/covariate data and blood/urine samples were obtained through family/household visits (TNFDS - Chidambaram/Karaikudi and vicinities and JFDS - Jaipur) and clinic visits (NFDS - Nellore) by the trained research/clinical staff members of each of the three FDRCs during the years 2016-2018. In regard to the TNFDS, family members were recruited from the towns of Chidambaram and Karaikudi (straight line distance between the two towns is approximately 110 miles) and their surrounding areas. Anthropometric data including weight, height, waist and hip circumferences (i.e., average of three values collected for a given trait) were collected using standardized procedures (51). Body mass index (BMI) was measured as weight (kg) divided by height (m^2^). Systolic and diastolic blood pressure and heart rate (i.e., average of three values collected for a given trait) were measured using Omron HEM -8712 blood pressure monitor. Fasting (at least 8-hour overnight fast) and post prandial capillary blood glucose levels were measured using Accu-Chek instant S glucometer at the study sites. The serum and EDTA blood samples and urine samples collected from all the participants from study sites were transported on dry ice within 24 hrs to the DCC at SRIHER for processing and biochemical analysis in an accredited laboratory and storage of biospecimens for use in the future. All assays were performed using standardized procedures, and all data were screened through standard QC measures prior to data analyses. Fasting plasma total cholesterol, triglycerides, HDL-cholesterol, and LDL-cholesterol were measured based on enzymatic photometric method using AU680 Clinical chemistry analyzer (Beckman Coulter Inc, Indianapolis, US). Hemoglobin A1c (HbA1c) levels were measured based on the principle of high performance liquid chromatography method using automated D-10 hemoglobin testing system (Bio-Rad Laboratories Inc., Hercules, CA, US). Fasting serum insulin was quantified based on chemiluminescent immunoassay using DxH 800 hematology analyzer (Beckman Coulter Inc., Indianapolis, US). Serum and urine creatinine levels were determined by Jaffe’s method using AU5800 Clinical chemistry analyzer (Beckman Coulter Inc., Indianapolis, US). The high-sensitivity C-reactive Protein (hs-CRP) was measured using the AU5800 Beckman coulter system.

T2D was defined by fasting capillary blood glucose ≥ 126 mg/dl and/or HbA1c ≥ 6.5% (52). Participants who did not meet these criteria but who reported that they were under treatment with either oral antidiabetic agents or insulin and who gave a history of diabetes were also considered to have T2D. In addition, all non-T2D study participants were examined for the presence of prediabetes using the following criteria: fasting capillary blood glucose = 100-125 mg/dl and/or HbA1c = 5.7% - 6.4% (52). From the fasting glucose and insulin concentrations, we estimated insulin resistance using the homeostasis model assessment of insulin resistance (HOMA-IR) (53). Following the World Health Organization (WHO) Asia Pacific Guidelines (54) and the Phase I of the ICMR-INDIAB study on obesity (55), generalized obesity (GO) was defined as a BMI ≥ 25 kg/m^2^ for both genders and abdominal obesity (AO) as a waist circumference ≥ 90 cm for men and ≥ 80 cm for women with or without GO. A questionnaire was used to collect information on demographic and environmental factors and covariate data including household information, family history of diabetes, medical history of diabetes and related health conditions, medication status, smoking status, alcohol consumption, socioeconomic status, educational status, psychological or behavioral attributes, dietary intake, and physical activity based on standardized questionnaires (56–58).

### Statistical/Genetic Analysis

Different statistical techniques were used to analyze the data including descriptive statistics. Group differences between the three EEGs were examined using ANOVA (Continuous traits) or Chi-square test (Discrete traits); superscript letters a, b, and c were used to refer to homogenous groups identified by Bonferroni’s *post hoc* contrast; and, similarities were denoted by sharing the same letter. The hierarchical multiple logistic regression analysis was used to assess association between T2D and correlated factors (e.g., GO, Education, EEG) through odds ratio (OR) statistic. For this analysis, the combined sample of the three EEGs with blocks (i.e., Block 1 = sex, age/groups, and EEG; Block 2 = obesity types (e.g., GO); and Block 3 = Socioeconomic status, Education status, Smoking status, and Alcohol consumption status) was used. All variables were analyzed as dummy variables. Block 1 variables were held constant, and Blocks 2 and 3 were analyzed using the backward elimination procedure (P-values for entry and retention were 0.05 and 0.10, respectively). All analyses were carried out using IBM^®^ SPSS.

The heritabilities of T2D and its related traits were determined using a variance components (VC) approach as implemented in the program SOLAR. To address the issue of non-normality, all quantitative traits were transformed using inverse normal transformation. In a simple model, variances or covariances between relatives as a function of the genetic relationships were specified, and the proportion of phenotypic variance that is attributed to (additive) genetic effects (i.e., heritability: h^2^) was estimated from the components of variance (59, 60). For such a model, the covariance matrix for a family (Ω) is given by: $\Omega =2\Phi \sigma_{\text{g}}^{2}+\text{I}\sigma_{\text{e}}^{2}$ , where Φ is the kinship matrix, $\sigma_{\text{g}}^{2}$ is the genetic variance due to additive genetic effects, I is the identity matrix, and $\sigma_{\text{e}}^{2}$ is the variance due to individual-specific environmental effects. A likelihood ratio test was used to test whether the heritability of a given trait is significant (P ≤ 0.05). Covariates (i.e., age, sex, age x sex, age^2^, age^2^ x sex, or BMI [T2D analysis only] were included in all analyses if found to be significant (P ≤ 0.10). This method was extended to the dichotomous traits such as T2D, using a threshold or liability model (61). Given our family ascertainment scheme, all genetic analyses were performed by correcting for the ascertainment, as described previously (62). All genetic analyses were performed using the computer program SOLAR (63).

## Results

Given the goal of enrolling 1,500 individuals from the three studies, we actually recruited 1,547 individuals from the three study sites: TNFDS = 517 (518 individuals were recruited, but one individual with type 1 diabetes was excluded from all analyses) from 21 families; JFDS = 530 from 25 families; and NFDS = 500 from 22 families (**Table 1**). The CH, AG, RE, and SI abbreviations are used from here forth to refer to the EEGs of Chettiars (TNFDS), Agarwals (JFDS), Reddys (NFDS), and Sikhs (AIDHS/SDS; for the purpose of comparison based on available data), respectively. The average family size in the combined sample of the three EEGS (i.e., CH, AG, and RE) is ~23, which ranged from 12-41. The characteristics of the study participants by EEG/FDRC for the demographic and selected T2D and its related glycemic and other cardiometabolic traits for this study are shown in **Table 1**, including prediabetes, generalized obesity (GO), abdominal obesity (AO), BMI, waist circumference (WC), fasting capillary blood glucose (FG), HOMA-IR, fasting insulin (FI), HbA1c, total cholesterol (TCHOL), triglycerides (TG), LDL-cholesterol (LDL-C), HDL-cholesterol (HDL-C), systolic (SBP) and diastolic (DBP) blood pressure, and high-sensitivity CRP (hs-CRP).

**Table 1:** Characteristics of the participants of TNFDS, JFDS, and NFDS.

Variable^@^ EEG (FDRC, N)^#^	All FDRCs (N = 1,547)	Chettiar (CH)TNFDS (N = 517)	Agarwal (AG)JFDS (N = 530)	Reddy (RE)NFDS (N = 500)	P-value^
Number of families	68	21	25	22	–
Average family size	23	25	21	23	–
Family size range	12-41	14-41	12-35	12-39	–
Age (years)	45.48 (16.37)	47.18^a^ (16.38)	43.41^b^ (16.44)	45.92^a,b^ (16.09)^^^	<0.001
Age of T2D onset (years)	48.57 (12.25)	50.41 (11.55)	47.35 (11.59)	48.16 (13.40)	*0.051*
BMI (kg/m^2^)	26.70 (5.19)	26.81 (5.29)	26.83 (4.70)	26.45 (5.55)	*0.420*
Waist circumference [WC] (cm)	95.4 (13.23)	97.98^a^ (13.69)	93.36^b^ (11.96)	94.89^b^ (13.60)	<0.001
Fasting blood glucose [FG] (mg/dL)^$^	119.3 (42.72)	125.5^a^ (51.29)	118.5^b^ (29.76)	113.6^b^ (43.85)	<0.001
HOMA-IR^$^	3.41 (7.42)	2.89^b^ (2.69)	4.44^a^ (8.10)	2.84^b^ (9.58)	<0.001
Fasting Insulin [FI] (IU/ml)^$^	10.97 (17.47)	9.22^b^ (6.75)	14.29^a^ (22.18)	9.27^b^ (18.96)	<0.001
HbA1c (%)^$^	6.40 (1.73)	6.34 (1.81)	6.52 (1.64)	6.32 (1.75)	*0.110*
Total cholesterol [TCHOL] (mg/dL)	161.1 (46.52)	180.5^a^ (40.92)	145.0^c^ (48.44)	158.2^b^ (42.56)	<0.001
Triglycerides [TG] (mg/dL)	125.9 (80.41)	136.0^a^ (86.65)	118.2^b^ (70.13)	123.6^b^ (82.95)	0.001
LDL-Cholesterol [LDL-C] (mg/dL)	100.8 (35.53)	116.2^a^ (33.45)	86.88^c^ (34.89)	99.77^b^ (31.78)	<0.001
HDL-Cholesterol [HDL-C] (mg/dL)	43.98 (13.94)	47.13^a^ (12.26)	40.55^c^ (15.97)	44.36^b^ (12.38)	<0.001
Systolic blood pressure [SBP] (mmHg)	128.0 (18.79)	132.6^a^ (18.66)	125.5^b^ (18.43)	125.9^b^ (18.46)	<0.001
Diastolic blood pressure [DBP] (mmHg)	81.98 (11.41)	82.80^a^ (11.28)	82.42^a^ (10.74)	80.67^b^ (12.13)	0.007
High-sensitivity CRP [hs-CRP] (mg/L)	0.44 (0.79)	0.51 (1.02)^a^	0.35 (0.47)^b^	0.45 (0.79)^a^	<0.001
Sex (F/M, %F)	761/786 (49)	267/250^a^ (52)	217/313^b^ (41)	277/223^a^ (55)	<0.001
T2D (n,%)	543 (35)	169 (33)	196 (37)	178 (36)	*0.333*
Prediabetes (n,%)	606 (60)	228^a^ (66)	217^a^ (65)	161^b^ (50)	<0.001
Generalized obesity (GO; n/%)	957 (62)	311^a,b^ (60)	353^b^ (67)	293^a^ (59)	*0.190*
Abdominal obesity (AO; n/%)	1257 (81)	445^a^ (86)	406^b^ (77)	406^a,b^ (81)	<0.001
Education Status (n, %)					
Uneducated	102 (6.59)	5^a^ (0.96)	2^a^ (0.37)	95^b^ (19)	<0.001
School	675 (43.6)	268^a^ (51.8)	133^b^ (25.0)	274^a^ (54.8)	
Graduate	576 (37.2)	180^a^ (34.8)	298^b^ (56.2)	98^c^ (19.6)	
Postgraduate	194 (12.5)	64^a^ (12.3)	97^b^ (18.3)	33^c^ (6.6)	
Socioeconomic Status (n, %)					
Lower	29 (1.87)	3^a^ (0.58)	0^a^ (0)	26^b^ (5.2)	<0.001
Middle	1314 (84.9)	491^a^ (94.9)	383^b^ (72.2)	440^c^ (88)	
Upper	204 (13.1)	23^a^ (4.44)	147^b^ (27.7)	34^a^ (6.8)	
Smoking Status (n, %)					
Never	1382 (89.3)	495^a^ (95.7)	433^b^ (81.7)	454^c^ (90.8)	<0.001
Former	51 (3.3)	7^a^ (1.4)	22^b^ (4.2)	22^b^ (4.4)	
Current	114 (7.4)	15^a^ (2.9)	75^b^ (14.2)	24^a^ (4.8)	
Alcohol Consumption Status (n, %)					
Never	1393 (91.3)	493^a^ (95.4)	436^b^ (85.7)	464^a^ (92.8)	<0.001
Former	30 (2)	5^a^ (1)	10^a^ (2)	30^a^ (3)	
Current	103 (6.7)	19^a^ (3.7)	63^b^ (12.4)	21^a^ (4.2)	

^#^EEG, Endogamous ethnic group; FDRC, Family Diabetes Research Center; N, Sample size; ^@^BMI, Body Mass Index; HOMA-IR, Homeostatic Model Assessment of Insulin Resistance; HbA1c, Hemoglobin A1c; High-sensitivity CRP (hs-CRP), High-sensitivity C-Reactive Protein. Continuous variables are expressed as mean (standard deviation) and Discrete variables are shown as counts and percentages (n, %), Group differences are tested using Analysis of variance (Continuous traits) or Chi-square test (Discrete traits) - superscript letters a, b, and c refer to homogenous groups identified by Bonferroni’s post hoc contrast and similarities are denoted by sharing the same letter. Please see text for trait definitions; ^^^Includes one participant aged 15 years; ^Non-significant differences (i.e., P > 0.05) are shown in italics, and P-values are not corrected for non-independence; ^$^Glycemic traits information including individuals with T2D (on medication) is provided for the purpose of comparison; however, their information related to non-T2D individuals is provided in **Table 2**.

The characteristics of the participants of TNFDS (CH), JFDS (AG), and NFDS (RE) are reported in **Table 1**. A number of variables exhibited significant differences (P ≤ 0.05) between the EEGs, while the differences regarding traits such as T2D prevalence, age of T2D onset, BMI, and HbA1c were found to be non-significant. As can be seen, above 50% of the study participants from CH and RE EEGs were females, while 41% of the AG sample were females. Based on average ages, the AG sample was relatively younger (43 y) compared to CH (47 y) and RE (46 y), respectively. The prevalence of T2D was found to be high, ranging from 33% (CH) to 37% (AG), while the average BMI among the three EEGs was found to be more or less similar (~27). The prevalence rates of obesity and prediabetes were also found to be high among the three EEGs. The differences in GO (range: 59%-67%) between EEGs were not significant, while AO exhibited significant differences between EEGs ranging from 77% (AG) to 86% (CH). The prevalence rates of prediabetes differed significantly among EEGs (range: 50% [RE]-66% [CH]). Although the three EEGs differed from each other regarding a number of T2D-related traits, certain EEG pairs exhibited non-significant differences or similarities in regard to certain traits (**Table 1**). For example, CH and RE contrasted with AG in regard to FI, HOMA-IR, and hs-CRP, while CH contrasted with AG and RE regarding WC, FG, TG, and SBP.

As reported in **Table 1**, significant differences were found regarding education, socioeconomic, smoking, and alcohol consumption statuses among the three EEGs. For example, more than 56% of AGs had graduate level education compared to 35% in CH and 20% in RE, respectively, while 19% of RE sample were uneducated versus less than 1% uneducated individuals from CH and AG EEGs. A majority of the families from the three EEGs were reported to belong middle socioeconomic status (range: 72% [AG] - 95% [CH]), and about 28% of AGs were found to belong to upper socioeconomic status compared to 4% (CH) and 7% (RE), respectively. The smoking and alcohol consumption behaviors (males only) were largely absent in the three EEGs. However, there were more smokers (former/current) in AG (4.0%/14.2%) and RE (4.4%/4.8%) compared to CH (1.4%/2.9%) and more alcohol drinkers (former/current) in AG (2%/12%) compared to RE (3%/4%) and CH (1%-4%), respectively.

The results from hierarchical logistic regression analysis of the combined sample of the three EEGs including the significant predictors of T2D are shown in **Table 2**. As can be seen, the occurrence of T2D was more in males and T2D’s risk is increased by age category in a stepwise fashion in reference to the age group 24 years and below. For example, based on odds ratio (OR), individuals in age group 35-44 years are approximately 7 times more likely to be T2D, while those in age group 65 and above years are approximately 42 times more likely to have T2D. In reference to CH, AG EEG is almost 2 times more likely to have T2D. AO is a strong correlate of T2D; individuals with AO are 2 times more likely to have T2D. Of the demographic and habitual behavioral traits considered, former smokers are more than 2 times likely to be affected with T2D.

**Table 2:** Hierarchical logistic regression analysis with blocks of significant predictor variables of type 2 diabetes in the combined data sets of TNFDS, JFDS, and NFDS.

Block/Variables^#^	β (SE)	OR (95% CI)	P-value^@^
Block 1: Sex (Male)	0.30 (0.13)	1.35 (1.04, 1.75)	0.0260
** **Age groups			
24 and below	–	–	–
25-34	0.83 (0.45)	2.29 (0.95, 5.54)	0.0650
35-44	1.90 (0.41)	6.71 (2.98, 15.13)	< 0.0001
45-54	2.87 (0.41)	17.61 (7.94, 39.02)	< 0.0001
55-64	3.51 (0.41)	33.43 (14.96, 74.69)	< 0.0001
65 and above	3.73 (0.42)	41.59 (18.30, 94.55)	< 0.0001
** **EEG			
CH	–	–	
AG	0.47 (0.16)	1.61 (1.18, 2.18)	0.0020
RE	0.25 (0.15)	1.29 (0.96, 1.73)	0.0960
Block 2: AO	0.69 (0.19)	2.00 (1.38, 2.92)	0.0003
Block 3: Smoking status			
Never	–	–	–
Former	0.77 (0.35)	2.15 (1.08, 4.31)	0.0300
Current	0.38 (0.24)	1.46 (0.91, 2.33)	0.1140

^#^Hierarchical logistic regression model included three blocks: Block 1, Sex, age, and EEG; which were held constant; Block 2, GO and AO, which were adjusted for sex. In addition, combined obesity (i.e., GO + AO) was also included as a variable for model efficiency; Block 3, Socioeconomic status, Education status, Smoking status, and Alcohol consumption status. All variables were analyzed as dummy variables. Blocks 2 and 3 were analyzed using the backward elimination procedure and the significance thresholds considered for entry and retention were 0.05 and 0.10, respectively; ^@^P-values are not corrected for non-independence.

The characteristics of selected traits by T2D status and EEG and the findings of group differences between EEGs are reported in **Table 3**. For the purpose of comparison, the SI data are included. In general, the trait differences between T2D and non-T2D individuals within a given EEG were found to be as expected. For example, FG means of T2D individuals ranged from 147.2 mg/dl (AG) to 188.6 mg/dl (SI), and CH and SI FG profiles differed from both AG and RE, respectively. In non-T2D individuals, it ranged from 93.74 mg/dl (RE) to 102.2 mg/dl (CH), and CH and AG as homogenous groups differed from homogenous groups of RE and SI. In regard to mean TG values, it ranged from 144.3 mg/dl (AG) to 187.9 mg/dl (SI) in individuals with T2D; although SI shares similarity with CH, it differs from AG and RE in mean TG profiles, respectively. In non-T2D individuals, mean TG values ranged from 102.8 mg/dl (AG) to 158.9 mg/dl (SI); SI exhibits its distinction from the other three groups; and RE aligns with both CH and AG groups, although CH and AG fail to be homogenous groups. The mean FI values in non-T2D individuals ranged from 7.39 (IU/ml) (RE) to 12.24 (IU/ml) (SI); the AG and SI groups are found to be homogenous in their mean FI profiles and they differed from both CH and RE, respectively.

**Table 3:** Comparison of selected traits by T2D status and EEG.

Variables^@^ EEG^#^	CH	AG	RE	SI^$^
**A. T2D Individuals**				
Age (years)	57.19 (13.24)	55.94 (12.50)	54.23 (14.26)	53.8 (11.4)
Age of T2D onset (years)	50.41^a^ (11.55)	47.35^a,b^ (11.59)	48.16^a,b^ (13.40)	46.1^b^ (10.7)
BMI (kg/m^2^)	27.77 (4.88)	27.76 (4.66)	27.10 (5.90)	27.5 (4.7)
WC (cm)	100.3^a^ (12.84)	97.93^a^ (11.28)	98.26^a^ (13.05)	94.4^b^ (10.9)
SBP (mmHg)	139.7^b^ (20.23)	133.6^c^ (19.56)	132.6^c^ (18.72)	149.82^a^ (23.81)
DBP (mmHg)	84.48^a,b^ (12.34)	85.17^a,b^ (11.42)	82.68^b^ (10.69)	86.37^a^ (11.66)
FG (mg/dl)	173.5^a^ (65.97)	147.2^c^ (31.22)	149.8^b^ (56.32)	188.6^a^ (72.1)
HbA1c (%)	8.11^a,b^ (2.242)	8.10^a,b^ (1.71)	7.90^b^ (2.046)	8.8^a^ (2.4)
TCHOL (mg/dl)	185.9^a^ (44.38)	141.3^c^ (48.98)	164.2^b^ (45.98)	181.2^a^ (45.7)
TG (mg/dl)	169.2^a,b^ (108.8)	144.3^b^ (84.31)	146.0^b^ (101.9)	187.9^a^ (106.6)
LDL-C (mg/dl)	116.6^a^ (37.85)	82.31^c^ (35.42)	103.1^b^ (34.57)	104.2^a,b^ (37.6)
HDL-C (mg/dl)	47.02^a^ (12.62)	38.73^b^ (15.78)	44.56^a^ (14.90)	39.5^b^ (12.6)
**B. Non-T2D Individuals**				
Age (years)	42.31^b^ (15.55)	36.06^c^ (13.83)	41.33^b^ (15.19)	46.2^a^ (14.7)
BMI ((kg/m^2^)	26.34 (5.422)	26.28 (4.65)	26.08 (5.32)	27.2 (4.7)
WC (cm)	96.82^a^ (13.95)	90.66^b^ (11.54)	93.02^b^ (13.54)	91.7^b^ (11.7)
SBP (mmHg)	129.1^b^ (16.82)	120.7^c^ (15.95)	122.2^c^ (17.27)	137.71^a^ (23.30)
DBP (mmHg)	81.99^a,b^ (10.65)	80.80^a,b^ (9.991)	79.56^b^ (12.74)	83.26^a^ (12.15)
FG (mg/dl)	102.2^a^ (11.75)	101.7^a^ (8.25)	93.74^b^ (11.15)	96.2^b^ (10.7)
HbA1c (%)	5.49^b,c^ (0.42)	5.59^b^ (0.430)	5.44^c^ (0.524)	6.43^a^ (1.7)
FI (IU/ml)	9.31^b^ (7.21)	11.53^a^ (11.53)	7.39^c^ (5.91)	12.24^a^ (10.41)
HOMA-IR	2.37^b^ (1.93)	2.93^a^ (3.03)	1.72^c^ (1.46)	2.4^a,b^ (2.1)
TCHOL (mg/dl)	177.9^a^ (38.93)	147.1^b^ (48.07)	154.9^b^ (40.24)	175.7^a^ (43.3)
TG (mg/dl)	120.0^b^ (68.04)	102.8^c^ (54.86)	111.2^b,c^ (67.31)	158.9^a^ (78.9)
LDL-C (mg/dl)	116.0^a^ (31.14)	89.56^c^ (34.34)	97.91^b^ (30.02)	100.1^b^ (33.8)
HDL-C (mg/dl)	47.18^a^ (12.09)	41.61^c^ (16.01)	44.25^b^ (10.75)	41.0^c^ (10.9)

^#^EEG, Endogamous ethnic group; ^$^SI/AIDHS/SDS, Sikh EEG representing the Asian Indian Diabetic Heart Study/Sikh Diabetes Study (data were previously collected and used here for the purpose of comparison; please see text for references); N, 1,260; Families, 324 (families were ascertained on multiple siblings with T2D), Average family size, 6.7; Family size range, 2-59; Females (%), 38.5%; T2D (%), 74.7; ^@^T2D, Type 2 diabetes; non-T2D, nondiabetics; BMI, Body Mass Index; WC, Waist circumference; SBP, Systolic blood pressure; DBP, Diastolic blood pressure; FG, Fasting blood glucose; HbA1c, Hemoglobin A1c; FI, Fasting insulin; HOMA-IR, Homeostatic Model Assessment of Insulin Resistance; TCHOL, Total cholesterol; TG, Triglycerides; LDL-C, LDL-cholesterol; HDL-C, HDL cholesterol. All variables are expressed as mean (standard deviation) and group differences are tested using Analysis of variance - superscript letters a, b, and c refer to homogenous groups identified by Bonferroni’s post hoc contrast (P < 0.001) and similarities are denoted by sharing the same letter. P-values are not corrected for non-independence. Please see text for trait definitions. Values of FI and HOMA-IR for individuals with T2D are not shown in the table given the potential impact of medication on their concentrations. However, HbA1c and FG values for diabetics are shown for depicting the glycemic status and profile comparisons across the EEGs, respectively. Please note that the variables reported in the table were not adjusted for any medication influences.

Since T2D and its related traits are complex phenotypes that are influenced by genetic and environmental factors, we determined heritability (i.e., h^2^ = the proportion of phenotypic variation in a given trait attributable to additive genetic influences) estimates for selected traits using family data from CH, AG, and RE, respectively. For the purpose of comparison, the already available heritability estimates for T2D and a few T2D related traits using SI family data are also reported. The number of families and related information from each EEG are provided in **Tables 1** (CH, AG, and RE) and 3 (SI). The types and numbers of relative pairs among study participants by EEG are reported in **Table 4**. The total number of relative pairs generated from each of the four EEGs family data sets are as follows: CH = 2,899, RE = 2,477, AG = 5,456, and SI = 2,393.

**Table 4:** Types and numbers of relative pairs among study participants by EEG.

Type of Relative Pair	Number of Pairs			
	CH	RE	AG	SI
Parent-Offspring	269	318	369	480
Siblings	234	180	407	833
Grandparent-Grandchild	46	90	111	17
Avuncular	343	329	1,021	337
Grand Avuncular	71	97	397	19
1^st^ Cousins	365	369	1,115	347
1^st^ Cousins, 1 rem	381	473	1,247	186
2^nd^ Cousins	375	314	510	49
2^nd^ Cousins, 1 rem	354	150	141	29
3^rd^ Cousins	159	38	–	6
3^rd^ Cousins, 1rem	41	4	–	–
Others	261	115	138	90
**Total**	**2,899**	**2,477**	**5,456**	**2,393**

The heritability estimates obtained from CH, AG, and RE family data sets after accounting for ascertainment correction (excluding traits from non-T2D individuals only) are reported in **Table 5**. All quantitative traits were transformed using inverse normal transformation for genetic analysis and T2D was analyzed as a dichotomous trait (i.e., liability model) using the VC approach. All traits were adjusted for age and sex terms as stated earlier if found to be significant. T2D was analyzed with and without BMI as a covariate in addition to adjustment for age and sex terms. All h^2^ estimates for the selected traits by EEG reported in **Table 5** were found to be significant (P ≤ 0.05), excluding SBP and DBP in the RE sample. The h^2^s for T2D by EEG are as follows: CH = 0.30 (30%), AG = 0.82 (82%), and RE = 0.46 (46%). For the purpose of comparison, it was found to be 54% in SI (**Table 5**; h^2^ estimates for FG, HDL-C, and LDL-C are also available as shown for the purpose of comparison). Additional adjustment for BMI in the T2D analyses yielded more or less similar h^2^ estimates. For the remaining traits, significant h^2^ estimates ranged from 25% (hs-CRP) to 81% (FI; non-T2D only) in CH, from 17% (TG) to 86% (FG; non-T2D only) in AG, and from 17% (hs-CRP) to 68% (HbA1c; non-T2D only) in RE, respectively. Overall, the T2D and its related traits in AG, CH, and RE EEGs exhibited strong genetic influences (h^2^ range: 17% [TG/AG and hs-CRP/RE] - 86% [FG/non-T2D/AG]).

**Table 5:** Heritability estimates for the selected traits by EEG.

Variable EEG	CH			AG			RE		
	N^#^	h^2^ ± SE	*P*-value	N^#^	h^2^ ± SE	*P*-value	N^#^	h^2^ ± SE	P-value^
T2D	517	0.30 ± 0.20	0.0466	530	0.82 ± 0.25	0.0003	500	0.46 ± 0.17	0.0028
T2D_adjBMI^@^	517	0.29 ± 0.17	0.0455	530	0.83 ± 0.25	0.0002	500	0.48 ± 0.18	0.0024
BMI	517	0.53 ± 0.09	1.4 x 10^-10^	530	0.47 ± 0.09	3.1 x 10^-10^	500	0.44 ± 0.09	2.0 x 10^-07^
WC	517	0.51 ± 0.09	1.3 x 10^-09^	530	0.46 ± 0.10	1.9 x 10^-09^	500	0.61 ± 0.10	1.6 x 10^-09^
HDL-C	517	0.65 ± 0.09	2.9 x 10^-16^	530	0.71 ± 0.08	6.1 x 10^-28^	500	0.49 ± 0.09	4.0 x 10^-10^
LDL-C	517	0.36 ± 0.09	3.0 x 10^-07^	530	0.36 ± 0.08	2.3 x 10^-10^	500	0.49 ± 0.08	1.1 x 10^-10^
TG	517	0.37 ± 0.08	7.9 x 10^-09^	530	0.17 ± 0.10	0.0217	500	0.27 ± 0.11	0.0026
TCHOL	517	0.39 ± 0.08	1.4 x 10^-08^	530	0.36 ± 0.08	1.6 x 10^-09^	500	0.50 ± 0.09	6.2 x 10^-11^
SBP	517	0.31 ± 0.09	0.0001	530	0.39 ± 0.09	9.5 x 10^-09^	500	0.11 ± 0.10	*0.1289*
DBP	517	0.26 ± 0.10	0.0007	530	0.38 ± 0.08	4.6 x 10^-11^	500	0.13 ± 0.09	*0.0578*
hs-CRP	517	0.25 ± 0.09	4.9 x10^-04^	530	0.33 ± 0.09	1.0 x 10^-06^	500	0.17 ± 0.09	0.0156
FG (non-T2D)^$^	348	0.43 ± 0.14	0.0004	334	0.86 ± 0.11	1.1 x 10^-15^	322	0.66 ± 0.11	2.3 x 10^-09^
FI (non-T2D)^$^	348	0.81 ± 0.11	2.0 x 10^-11^	334	0.32 ± 0.13	0.0020	322	0.43 ± 0.15	0.0011
HOMA-IR (non-T2D)^$^	348	0.74 ± 0.11	1.5 x 10^-09^	334	0.33 ± 0.13	0.0023	322	0.37 ± 0.15	0.0051
HbA1c (non-T2D)^$^	348	0.39 ± 0.14	0.0016	330	0.64 ± 0.12	2.4 x 10^-09^	322	0.68 ± 0.13	1.0 x 10^-07^

^#^All quantitative traits were transformed using inverse normal transformation and adjusted for age and sex terms (i.e., age, sex, age x sex, age^2^, age^2^ x sex) if found to be significant for genetic analyses; ^@^T2D (discrete trait) was analyzed with and without BMI as a covariate, in addition to age and sex terms as covariates; ^$^Genetic analysis of glycemic traits were based on data from non-T2D individuals only; ^Heritability estimates that were not significant (i.e., P > 0.05) are shown in italics. For the purpose of comparison, h^2^ estimates for selected traits from SI are as follows: T2D = 54%, FG = 54%, HDL-C = 87% and LDL-C = 44%.

## Discussion

In this study, we aimed to assess the burden of T2D and its related traits using family data collected from three EEGs (i.e., Chettiar/CH, Agarwal/AG, and Reddy/RE) with the same study design and methodological tools, representing three different languages and geographical locations in India, as part of the Indo-US (ICMR/NIH) joint collaborative research projects related to diabetes. In addition to these newly collected data, for the purpose of comparison, we used already available data from our other family study representing a linguistically and geographically distinct population of Khatri Sikhs (SI). Aside from depicting the prevalence and familial aggregation (i.e., clustering of diseases or traits within families due to genetic and/or environmental similarities) profiles of T2D and associated cardiometabolic traits, we determined the extent to which variation in a given trait is due to additive genetic influences using family data. These family studies represent our INDIGENIUS consortium studies (**Figure 2**).

The CH, AG, and RE EEGs bear substantial T2D and its related clinical burdens (**Table 1**). The T2D prevalence rates were high given its familial aggregation, which ranged from 33% (CH) to 37% (AG) (Overall prevalence = 35%), and the differences between them were found to be not statistically different despite the fact that they represented distinct linguistic and geographical affiliations. The proportion of newly diagnosed T2D in the total T2D sample by EEG is as follows: CH = 28%, RE = 41%, and AG = 18%. However, the three EEGs are mostly representative of the middle socioeconomic status and urban (AG)/semiurban (CH and RE) communities. Recently, using data from a family-based study of the Sindhi endogamous population, the prevalence of T2D without and with adjustment for the ascertainment criteria was found to be ~30% and 35%, respectively, which are very similar to the prevalence rates observed in our study (37). Given attention to the differences in the T2D diagnostic criteria used by different studies, these prevalence rates are much higher than those reported for population-based studies in India such as the ICMR-INDIAB study because of the family-based nature of our studies and their ascertainment strategy. Hence, comparisons with other studies should be made with caution. Several studies have shown increased prevalence of T2D in urban areas compared to rural areas in India (3, 15, 64–68). For example, given that the CH and RE were recruited from the states of Tamil Nadu and Andhra Pradesh, the estimated prevalence rates of T2D in the urban (vs. rural) areas of the same states by the ICMR-INDIAB national population-based cross-sectional study were 13.7% (vs. 7.8%) and 12.6% (vs. 6.3%), respectively (3, 15). In a survey of 11 cities including Jaipur, the location of AG population in our study, the prevalence of T2D was found to be 15.7% (67) in middle class participants, which is comparable to other prevalence rates reported in the urban areas of Tamil Nadu (15.5%) and Andhra Pradesh (15.1%) states, respectively (66, 68). In another study involving an Urban population from Tamil Nadu, the occurrence of T2D increased along with ascending social class (Low = 12.0%, Middle = 18.4%, and High = 21.7%) (17). Also, our findings are compared to the age-adjusted prevalence rates of T2D reported for the US ethnic groups as part of the Mediators of Atherosclerosis in South Asians Living in America (MASALA) study and the Multi-Ethnic Study of Atherosclerosis (MESA) as follows: South Asians (23%), European Americans (6%), African Americans (18%), Latinos/Hispanics (17%), and Chinese Americans (13%) (69). In consideration of the above discussion, the prevalence of T2D found in the current study mirrors the substantial burden of T2D and its aggregation among Indian families.

To assess the high risk groups for T2D development, we estimated the prevalence of prediabetes using the ADA criteria. Its prevalence estimates ranged from 50% (RE) to 66% (CH) (Overall prevalence = 60%), and exhibited significant differences. Given attention to the issues such as the diagnostic criteria of prediabetes used, the choice of test, and the population being examined, the prevalence estimates across populations including those from India have been shown to vary greatly (70–75). According to the ICMR-INDIAB national data based on information from 15 states, the overall prevalence of prediabetes was estimated to be 10.3%, and its occurrence (i.e. urban vs. rural) in the States of Tamil Nadu, Andhra Pradesh, and Rajasthan was reported to be 9.8% vs. 7.1%, 11.1% vs. 9.6%, and 17.1% vs. 14.7%, respectively (3, 15, 76). However, the fasting glucose cutoffs used (i.e., ADA vs. WHO) to define impaired fasting glucose in the ICMR-INDIAB study national data resulted in a remarkable difference in the prevalence rates of both impaired fasting glucose and prediabetes: 20.8% vs. 6.5% and 24.7% vs. 10.3%, respectively (3). The familial aggregation profiles of prediabetes observed in our study are worrisome given the alarming rise in incidence rates of T2D and prediabetes based on longitudinal data in Indian populations as well as those from the MASALA Study (77–79).

The prevalence rates of generalized (GO: range = 59% [RE] – 67% [AG]), overall prevalence = 62%) and abdominal (AO: range = 77% [AG] – 86% [CH]), overall prevalence = 81%) obesity profiles observed in this study are disturbing taken together with the burden of T2D and prediabetes borne by the families within each EEG. Similar observations (i.e., prevalence of general obesity and central obesity was > 70%) were made in the Sindhi family study of T2D mentioned previously (37). As in the case of T2D, based on the national data (i.e., ICMR-INDIAB Study – Phase I), the occurrence of these obesity traits was high in urban areas compared to those from rural areas (55). For example, the prevalence rates of GO and AO in urban vs. rural areas in Tamil Nadu state were 35.7%, vs. 20.0%, and 37.4% vs. 22.1%, respectively. In another study from Chennai (urban), Tamil Nadu, the age standardized prevalence of GO (i.e., BMI ≥ 23 kg/m^2^) and AO were reported to be 45.9% and 46.6%, respectively (80). In a study from the state of Andhra Pradesh, the prevalence rates of GO and AO were 56.0% and 71.2%, respectively (81). Likewise, high prevalence rates of GO and AO were found in a New Delhi urban population, which were 50.1% and 68.9%, respectively (82). As revealed by the hierarchical logistic regression analysis of the combined sample of the three EEGs, in addition to sex (male), age (groups), EEG, and past smoking status, AO was determined to be a significant predictor of T2D (**Table 2**). It is known that AO is one of the major risk factors for T2D as well as cardiovascular disease in Asian Indians (83, 84). In addition to the above T2D, prediabetes, and obesity profiles, we examined differences between the EEGs regarding 13 quantitative traits related T2D, and only two of the 13 examined traits (i.e., BMI and HbA1c) failed to exhibit significant differences between the EEGs (**Table 1**). Based on selected traits and inclusion of data form the SI EEG for the purpose of comparison, the trait differences examined between T2D and non-T2D individuals within a given EEG were found to be as expected (**Table 3**). In general, the EEGs of CH and SI appear to have increased burden of lipid and blood pressure related conditions; however, based on information from non-T2D individuals, the EEGS of AG and SI appear to have distinct hyperinsulinemia/insulin resistance profiles.

Following the observed differential epidemiological profiles of T2D and related traits, we determined the extent to which these phenotypes are influenced by additive genetic influences using family data. Given that an estimate of heritability is population-specific, T2D and its related quantitative traits in the EEGs exhibited strong additive genetic influences. The heritabilities of T2D were found to be 30%, 46%, 54%, and 82% in CH, RE, SI, and AG EEGs, respectively, and statistically significant. The T2D heritability in the Sindhi family study was estimated to be 35% (37). The heritability estimates for the remaining T2D-related traits across the EEGs were significant, excluding SBP and DBP in the RE sample, which ranged from 25% (hs-CRP) to 81% (FI; non-T2D only) in CH, from 17% (TG) to 86% (FG; non-T2D only) in AG, and from 17% (hs-CRP) to 68% (HbA1c; non-T2D only) in RE, respectively. The available family based studies examined the occurrence of T2D among relatives to reflect shared genetic predisposition and the heritability profiles of T2D related traits in various Indian populations or Asian Indians, which differed in their study designs and analytical tools (26, 27, 35, 51, 85–89). For example, the heritabilities for selected traits for the purpose of discussion including fasting glucose, HbA1c, HDL-C, triglycerides, and systolic blood pressure were reported to be 24%, 36%, 39%, 22%, and 33% in the data obtained from multiplex families from Chennai, Tamil Nadu (26), and they were 37%, 60%, 53%, 40%, and 29% for the same traits in a subsample of the Asian Indian families from UK, respectively (35). For BMI, the heriatbilties were 44%, 31% and 25% in the above stated Chennai sample, UK sample, and a sample of selected EEGs including the Reddy EEG from Andhra Pradesh (51), respectively. In the Sindhi family study of T2D, heritability of anthropometric phenotypes ranged from 27% to 73%, while its range was 0% to 39% for T2D-related phenotypes (37). Following the above discussion, our genetic analyses of T2D and related traits revealed significant, substantial, and differential additive genetic influences on T2D and its related traits in the study samples. These findings set the scene for future studies to identify risk loci for the various cardiometabolic traits examined in this study using genome-wide association scans.

There are a few limitations of our study. We used capillary blood glucose estimates in our family- and community-based study for the purpose of comparison with our AIDHS/SDS study and the same procedure was used by some other population-based studies in India (3, 90). It has been shown that it is a feasible alternative to define T2D in epidemiological/population-based studies (3, 90–92). Moreover, T2D was defined in this study using information from HbA1c and/or use of medical records and diabetes medication/history in addition to fasting capillary blood glucose measures. Our methodological approach did not make an attempt to differentiate type 2 diabetes from type 1 diabetes or other types of diabetes based on any specific investigation, excepting exclusion of one individual with type 1 diabetes based on self-report/medical history as noted before.

In conclusion, our Indo-US exploratory/developmental collaborative study on T2D in Indian populations revealed high burden of T2D and its clinical correlates. In addition, these traits were found to be under substantial additive genetic influences in genetically and culturally diverse EEGs representing the northern and southern regions of India. Through comparisons with other populations, the Indian EEGs exhibited distinct T2D profiles underscoring the need for focused studies in near future with attention to genetic and sociocultural diversity of the Indian populations. Since the EEGs in this study are mostly representative of the middle socioeconomic status and urban/semiurban communities, there appears to be an immediate need to extend our approach to assess T2D burden in families of other diverse EEGs in India. Our efforts are reflective of feasibility of large-scale genetic studies of T2D through collaborations, both national and international. Given the worrisome T2D clinical profiles found in this study, it is imperative that aggressive public health awareness and preventive measures are implemented early on, and further suggesting the need for immediate plans for intervention studies. To be specific, individuals/families from the EEGs who participated in this study should be advised to start diabetes prevention measures early in life, either in adolescence or early youth, with greater focus on healthy diet, physical activity, and weight maintenance. There are numerous EEGs in India and similar studies could identify more EEGs where earlier intervention may be highly warranted.

These data also call for longitudinal assessments of the project participants to thoroughly understand the disease development and progression. In addition to our ongoing work on targeted sequencing of the GWAS-derived South Asian-specific T2D risk loci in the SI EEG sample and subsequent replication studies in the CH, AG, and RE EEGs, our immediate plans through potential future projects are 1) to generate omics data, especially whole genome sequencing data, from the family members of the diverse EEGs, current and new, representing various regions of India to understand the molecular basis of T2D in Indian populations; and 2) to conduct intervention studies (e.g., family-focused) that are culturally suited to Indian EEGs.

## Data Availability Statement

Given attention to the Institutional Ethics Committees policies and the INDIGENIUS Consortium data access policy, restrictions will apply to the availability of data used for this study publicly. However, data will be made available from the authors upon reasonable request, and data access requests should be submitted to the corresponding author.

## Ethics Statement

The study protocols involving human participants were reviewed and approved by the Institutional Ethics Committees of the Sri Ramachandra Institute of Higher Education and Research, Chennai, Tamil Nadu (Tamil Nadu Family Diabetes Study/TNFDS), the Fortis Escort Hospital, Jaipur, Rajasthan (Jaipur Family Diabetes Study/JFDS), and the Narayana Medical College and Hospital, Nellore, Andhra Pradesh (Nellore Family Diabetes Study/NFDS), India. Written informed consent for participation was obtained following the approvals by the three Institutional Ethical Committees. Already available family data from Phase I of the Asian Indian Diabetic Heart Study/Sikh Diabetes (AIDHS/SDS) were used for this study for the purpose of comparison. The analysis of de-identified data was approved by the Institutional Review Board, University of Texas Rio Grande Valley, Edinburg, Texas, USA. The patients/participants provided their written informed consent to participate in this study.

## Author Contributions

SP, RG, DK, TP, ST, KM, RV, DS, JB, and RD contributed to the study conception and study design. The clinical data and biospecimen sample collection, processing, and data management of TNFDS, JFDS, and NFDS were supervised by SP, CN, UR, RG, KM, DK, AP, and RD through direct contributions from VV, TK, DR, SS, SL, KS, MS, RR, PV, PR, NS, and JE. AIDHS/SDS data were collected and analyzed under the supervision of DS through direct interactions with CB, GW, SR, JS, and NM. VV, JL-A, RA, DR, MA, and RD performed the statistical analysis with contributions from SM, JB, SP, and DS regarding the data analysis and interpretation of the data. VV, JL-A, RA, and DR wrote the initial draft of the manuscript, which was critically revised by RD, SM, RG, DK, DS, and SP. All authors contributed to the article and approved the submitted version.

## Funding

This study is supported by grants from the Indian Council of Medical Research [ICMR] (India) Project: No. 55/6/2/Indo-US/2014-NCD-II and the National Institute of Diabetes and Digestive and Kidney Diseases [NIDDK], National Institutes of Health [NIH] (US) R21 DK105913 and R01 DK082766.

## Conflict of Interest

The authors declare that the research was conducted in the absence of any commercial or financial relationships that could be construed as a potential conflict of interest.

## Publisher’s Note

All claims expressed in this article are solely those of the authors and do not necessarily represent those of their affiliated organizations, or those of the publisher, the editors and the reviewers. Any product that may be evaluated in this article, or claim that may be made by its manufacturer, is not guaranteed or endorsed by the publisher.
